# Tryptophan Depletion Modulates Tryptophanyl-tRNA Synthetase-Mediated High-Affinity Tryptophan Uptake into Human Cells

**DOI:** 10.3390/genes11121423

**Published:** 2020-11-27

**Authors:** Takumi Yokosawa, Aomi Sato, Keisuke Wakasugi

**Affiliations:** 1 Department of Biological Sciences, Graduate School of Science, The University of Tokyo, 7-3-1 Hongo, Bunkyo-ku, Tokyo 113-0033, Japan; yokosawa@bio.c.u-tokyo.ac.jp; 2 Department of Life Sciences, Graduate School of Arts and Sciences, The University of Tokyo, 3-8-1 Komaba, Meguro-ku, Tokyo 153-8902, Japan; aoahiru061212@yahoo.co.jp

**Keywords:** aminoacyl-tRNA synthetase, tryptophanyl-tRNA synthetase, tryptophan uptake, amino acid transport, interferon-γ, indoleamine 2,3-dioxygenase, tryptophan 2,3-dioxygenase

## Abstract

The novel high-affinity tryptophan (Trp)-selective transport system is present at elevated levels in human interferon-γ (IFN-γ)-treated and indoleamine 2,3-dioxygenase 1 (IDO1)-expressing cells. High-affinity Trp uptake into cells results in extracellular Trp depletion and immune suppression. We have previously shown that both IDO1 and tryptophanyl-tRNA synthetase (TrpRS), whose expression levels are increased by IFN-γ, have a crucial function in high-affinity Trp uptake into human cells. Here, we aimed to elucidate the relationship between TrpRS and IDO1 in high-affinity Trp uptake. We demonstrated that overexpression of IDO1 in HeLa cells drastically enhances high-affinity Trp uptake upon addition of purified TrpRS protein to uptake assay buffer. We also clarified that high-affinity Trp uptake by Trp-starved cells is significantly enhanced by the addition of TrpRS protein to the assay buffer. Moreover, we showed that high-affinity Trp uptake is also markedly elevated by the addition of TrpRS protein to the assay buffer of cells overexpressing another Trp-metabolizing enzyme, tryptophan 2,3-dioxygenase (TDO2). Taken together, we conclude that Trp deficiency is crucial for high-affinity Trp uptake mediated by extracellular TrpRS.

## 1. Introduction

The novel tryptophan (Trp) transport system, which displays high affinity and selectivity for Trp, is highly expressed in human interferon-γ (IFN-γ)-treated or indoleamine 2,3-dioxygenase 1 (IDO1)-expressing cells [1,2,3,4]. Elevated levels of Trp uptake into cells leads to extracellular Trp depletion, causing immune suppression/immune tolerance [5,6,7,8,9,10,11,12,13,14,15,16]. Reduced availability of extracellular Trp blocks T cell proliferation, thereby providing a molecular basis for the immunosuppressive function [5,6,7,8,9,10,11,12,13,14,15,16].

IDO1 is a heme-containing enzyme that functions in a number of different organs, such as the placenta and immune cells. Expression of IDO1 is induced by IFN-γ, which coordinates a cascade of events to combat pathogens and neoplasia [17,18]. IDO1 catalyzes the first and rate-limiting step of the kynurenine (Kyn) pathway, which degrades Trp to Kyn. Moreover, IDO1 is thought to be involved in a number of biological roles, including regulatory immunity in fetal–maternal tolerance and control of antitumor immunity [5,6,7,8,9,10,11,12,13,14,15,16].

We have previously demonstrated that IDO1 and tryptophanyl-tRNA synthetase (TrpRS) are crucial for high-affinity Trp uptake [4]. IFN-γ induces the expression of human TrpRS but no other aminoacyl-tRNA synthetase [19,20,21,22,23,24,25]. In earlier work, we showed that IFN-γ treatment of human cells induces the expression of IDO1 and TrpRS, resulting in elevated levels of high-affinity Trp uptake into the cells [4]. We have also shown that siRNA-mediated inhibition of human TrpRS or IDO1 expression decreases high-affinity Trp uptake, whereas overexpression of human TrpRS or IDO1 increases high-affinity Trp uptake [4].

The ligation of amino acids to their cognate tRNAs is catalyzed by aminoacyl-tRNA synthetases [26]. The aminoacylation reaction proceeds in two distinct steps: (i) generation of an aminoacyl-AMP intermediate from the amino acid plus ATP, and then (ii) transfer of the aminoacyl moiety to a specific tRNA to give the aminoacyl-tRNA [27]. Using site-directed mutagenesis, we have previously revealed that the Trp- and ATP-binding pockets of human TrpRS, but not the tRNA-binding site, have a crucial role in the regulation of Trp uptake [4]. Consistent with this observation, noncanonical functions for TrpRS distinct from aminoacylation have been reported, including a role in cell-signaling pathways connected to the immune system and angiogenesis [4,28,29,30,31,32,33]. Furthermore, we have demonstrated that the addition of TrpRS protein to the assay buffer increases high-affinity Trp uptake into cells [4]. Given that human TrpRS is thought to be secreted from cells [32,34,35], we reasoned that extracellular TrpRS might regulate high-affinity Trp uptake. These observations highlight the importance of human TrpRS in the regulation of high-affinity Trp uptake.

Here, we investigated whether TrpRS and IDO1 have synergistic effects. We also analyzed the effects of Trp depletion on high-affinity Trp uptake by extracellular TrpRS. Finally, we examined the effects of tryptophan 2,3-dioxygenase (TDO2), a Trp-catabolizing enzyme that is expressed in tumor cells and induces immune resistance [36,37], on TrpRS-mediated high-affinity Trp uptake.

## 2. Materials and Methods

### 2.1. Reagents

Arginine (Arg), cysteine (Cys), histidine (His), isoleucine (Ile), leucine (Leu), lysine (Lys), methionine (Met), phenylalanine (Phe), serine (Ser), threonine (Thr), tyrosine (Tyr), valine (Val), kynurenine (Kyn), indole-3-carbinol (I3C) were obtained from Sigma-Aldrich (St. Louis, MO, USA). Glycine (Gly) and Trp were purchased from FUJIFILM Wako Pure Chemical Corporation (Osaka, Japan). L-[5-^3^H]Trp ([^3^H]Trp) was purchased from PerkinElmer (Waltham, MA, USA).

### 2.2. Plasmids

The cDNA encoding for human IDO1 (residue 1-403), purchased from RIKEN BioResource Center (Ibaraki, Japan), was engineered into the pcDNA3.1/myc-His(-) B mammalian expression vector [4]. The resulting gene product included a C-terminal tag of six consecutive histidine (6×His) residues [4]. A human TDO2 cDNA clone was obtained from TransOMIC Technologies (Huntsville, AL, USA). The coding sequence of human TDO2 (residue 1-406) was inserted into the pcDNA3.1 mammalian expression vector without any fusion tag. A cDNA fragment of human TrpRS (residue 1-471) was cloned into the prokaryotic expression vector pET20b (Merck Millipore, Darmstadt, Germany) so as to generate a gene product with a C-terminal tag of 6×His residues [4,33,38,39]. IDO1 and TDO2 mutant expression vectors were made by the QuikChange™ site-directed mutagenesis kit (Stratagene, La Jolla, CA, USA) to introduce base substitutions at specific sites. The anticipated sequences of the final constructs were verified (FASMAC Co. Ltd., DNA sequencing services, Atsugi, Japan) before proceeding further.

### 2.3. Preparation of Human TrpRS Protein

The pET20b expression construct encoding 6×His-tagged human TrpRS was used to transform *Escherichia coli (E. coli)* BL21(DE3) (Merck Millipore). The cells were grown at 37 °C to a low optical density (A_600_ of ≈0.8). Production of recombinant TrpRS was then induced by 0.4 mM isopropyl β-D-1-thiogalactopyranoside and cell culture was continued for a further 4 h before pelleting the bacteria. The recombinant 6×His-tagged TrpRS protein was subsequently purified to near homogeneity. Specially, the cell-free extract was applied to a nickel affinity column (His•Bind^®^ resin; Merck Millipore) according to the standard protocol described by the manufacturer. Endotoxin was effectively eliminated from the protein solution using EndotoxinOUT™ Resin (G-Biosciences, St. Louis, MO, USA) according to the manufacturer’s instructions. The level of endotoxin was <0.001 units/mL as estimated using a *Limulus* amebocyte lysate gel-clot assay (E-TOXATE kit; Sigma-Aldrich). The protein concentration of human TrpRS was determined using Bradford reagent (Bio-Rad laboratories Inc., Hercules, CA, USA) with bovine serum albumin (FUJIFILM Wako Pure Chemical Corporation) as standard.

### 2.4. Cell Line

Human HeLa cells (RCB0007) were obtained from the RIKEN Cell Bank (Tsukuba, Japan). The cell line was cultured in high glucose Dulbecco’s modified Eagle’s medium (DMEM) (Thermo Fisher Scientific Waltham, MA, USA, Catalog number: 10313-021), 10% (*v*/*v*) fetal bovine serum (FBS) (Biosera, Nuaille, France), and 2 mM glutamine (Gln) (FUJIFILM Wako Pure Chemical Corporation) and maintained under standard conditions in 5% CO_2_ at 37 °C.

### 2.5. Quantification of Trp Uptake

HeLa cells were washed three times in phosphate-buffered saline (PBS) (137 mM NaCl, 2.68 mM KCl, 8.1 mM Na_2_HPO_4_, 1.47 mM KH_2_PO_4_, pH 7.4), and resuspended in uptake assay buffer (PBS containing 300 µM MgCl_2_) at 1 × 10^6^ cells/mL. For the experiments conducted in sodium-free buffer, HeLa cells were washed three times in Tris-choline buffer (150 mM choline chloride (FUJIFILM Wako Pure Chemical Corporation), 10 mM Tris, pH 7.4) and resuspended in Tris-choline buffer at 1 × 10^6^ cells/mL. Then, 150 nM [^3^H]Trp was added to a 300 μL aliquot of resuspended cells in the absence or presence of 500 nM TrpRS protein. Trp uptake was then measured at specific time points (0, 2, 4, and 6 min). Measurements were conducted at 25 °C by subjecting 50 μL aliquots to rapid filtration. This was achieved by applying samples to Whatman^®^ GF/C glass microfiber filters (GE Healthcare Life Sciences, Buckinghamshire, England) on a vacuum filtration manifold (Merck Millipore). The filters were thoroughly washed five times with 5 mL PBS and air-dried. Then, the radioactivity of each sample was detected by scintillation counter (Tri-Carb 3180TR/SL low activity liquid scintillation analyzer, PerkinElmer). Trp uptake increased over time in a linear fashion. Initial uptake rate was determined by using the data at 0, 2, 4, and 6 min and expressed as fmol min^−1^ in these experiments.

### 2.6. Plasmid Transfection into HeLa Cells

HeLa cells were initially seeded at 1.5 × 10^5^ cells/mL using 60 mm Falcon^®^ tissue culture-treated dishes (Corning, Corning, NY, USA) one day prior to conducting the experiment. Transfections using polyethylenimine (PEI) “Max” (Polysciences, Warrington, PA, USA) were conducted for the pcDNA3.1/myc-His(-) B expression vector or empty vector (control). Typically, 5 μg plasmid DNA and 20 μL of PEI Max (1 mg/mL, pH 8.0) were mixed in 400 μL of Opti-MEM^®^ (Thermo Fisher Scientific, Waltham, MA, USA), incubated for 20 min at room temperature (≈25 °C), and added to the cells. The cells were incubated for 24 h after transfection.

### 2.7. Western Blotting

The protein samples were resolved on 12.0% SDS-polyacrylamide gels and electroblotted onto Immobilon^®^-P PVDF membranes (Merck Millipore), which were then blocked with 10 mM Tri-HCl, pH 8.0 containing 0.1% Tween 20 (Sigma-Aldrich) and 5% skimmed milk (FUJIFILM Wako Pure Chemical Corporation). Membranes were immersed for 1 h in PBS containing a mouse monoclonal antibody directed against the 6×His tag (Invitrogen), rabbit anti-IDO1 polyclonal antibodies (GeneTex, Irvine, CA, USA; Catalog number: GTX113753), rabbit anti-TDO2 polyclonal antibodies (GeneTex, Catalog number: GTX114831), mouse anti-β-actin monoclonal antibody (Cloud-Clone Corp., Houston, TX, USA), or rabbit anti-ATF4 polyclonal antibodies (GeneTex, Catalog number: GTX30070). After extensive washing with the buffer (10 mM Tris-HCl, 0.1% Tween 20, pH 8.0), the membranes were incubated with an HRP-linked secondary antibody (F(ab’)_2_ fragment of donkey anti-rabbit IgG or whole antibody of sheep anti-mouse IgG (GE Healthcare Life Sciences)) for 1 h. The membranes were subsequently washed three times with the buffer, and the proteins were visualized with ECL™ Western blotting detection reagents (GE Healthcare Life Sciences). A LAS-4000 mini Luminescent image analyzer (GE Healthcare Life Sciences) was used to acquire chemiluminescent signals.

### 2.8. Incubation of HeLa Cells in Trp-Free Medium

HeLa cells were seeded at 1.5 × 10^5^ cells/mL on Falcon^®^ tissue culture-treated 60 mm dishes (Corning). After 24 h, the cells were washed three times in PBS, and cultured in DMEM medium containing DMEM (high glucose) with sodium pyruvate but without amino acids (FUJIFILM Wako Pure Chemical Corporation, Catalog number: 048-33575), 10% (*v*/*v*) US dialyzed FBS (GE Healthcare Life Sciences), 2 mM Gln (FUJIFILM Wako Pure Chemical Corporation), and amino acids (0.4 mM Arg, 0.2 mM Cys, 0.4 mM Gly, 0.2 mM His, 0.8 mM Ile, 0.8 mM Leu, 0.8 mM Lys, 0.2 mM Met, 0.4 mM Phe, 0.4 mM Ser, 0.8 mM Thr, 0.4 mM Tyr, 0.8 mM Val). These concentrations of amino acids in this DMEM medium are the same as those in DMEM (Thermo Fisher Scientific, Catalog number: 10313-021) with the exception of 80 μM Trp, which is only included in DMEM (Thermo Fisher Scientific, Catalog number: 10313-021). The cells were incubated in the medium for 24 h.

### 2.9. Statistical Analyses

Data generated in this study were analyzed by one-way ANOVA followed by Tukey–Kramer post-hoc tests.

## 3. Results

### 3.1. Addition of Purified TrpRS Protein to the Assay Buffer Enhances Trp Uptake into HeLa Cells

**Figure 1:**
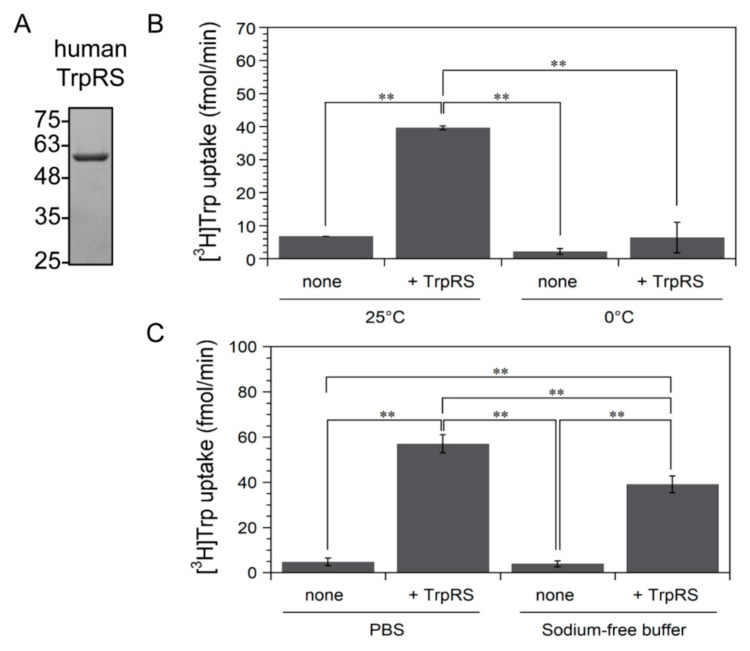
The effect of adding purified human tryptophanyl-tRNA synthetase (TrpRS) protein to the assay buffer on [^3^H]Trp uptake by HeLa cells. (**A**) SDS-PAGE analysis of purified human TrpRS protein. The gel was stained with Coomassie Brilliant Blue. Molecular size markers (kDa) are shown on the left. (**B**) [^3^H]Trp uptake at 25 or 0 °C. All data are given as mean ± standard deviation (SD) of three independent experiments. ** p < 0.01. (**C**) [^3^H]Trp uptake into cells in phosphate-buffered saline (PBS) or sodium-free Tris-choline buffer at 25 °C. All data are given as mean ± SD of three independent experiments. ** p < 0.01.

We previously showed that a novel Trp-selective transport system with high affinity for Trp is expressed in IFN-γ-treated cells [4]. Indeed, this system displays several hundred-fold higher affinity over that of the widely expressed neutral amino acid transporter System L, which transports Trp and other bulky hydrophobic amino acids [4]. In particular, we demonstrated that at low Trp concentrations (150 nM) the uptake of Trp relies on the novel Trp-selective transport system, which is regulated by TrpRS [4]. Here, we tried to investigate the effect of adding TrpRS protein to the assay buffer on the cellular high-affinity uptake of Trp. Recombinant human TrpRS was expressed in *E. coli* and purified, and its purity was confirmed by 12.0% SDS-polyacrylamide gel electrophoresis (SDS-PAGE) and Coomassie Brilliant Blue staining. A band corresponding to 6×His-tagged human TrpRS (predicted molecular mass: 55 kDa) was observed (Figure 1A). To facilitate quantification, [^3^H]Trp uptake into HeLa cells was measured. The addition of purified TrpRS protein to the assay buffer stimulated the cellular high-affinity Trp uptake (Figure 1B). Next, we tested the effect of lowering the temperature on high-affinity Trp uptake mediated by extracellular TrpRS. As shown in Figure 1B, lowering the temperature from 25 to 0 °C drastically suppressed Trp uptake. These results are very similar to those obtained using IFN-γ-treated cells at room temperature or 0 °C [4]. As membrane trafficking is drastically reduced at low temperature, our findings indicate that TrpRS-mediated high-affinity Trp uptake is facilitated by an energy-dependent transport mechanism rather than binding to a surface-receptor.

As previous reports have reported that amino acid transport systems are classified to Na^+^-dependent and Na^+^-independent systems [40,41] and that Trp-sensitive uptake does not require a transmembrane Na^+^ gradient [1], we next investigated the Na^+^ dependence of TrpRS-mediated high-affinity Trp uptake. As shown in Figure 1C, the increase of high-affinity Trp uptake upon addition of TrpRS protein was detected even in Na^+^-free buffer. This result agrees with previous data and suggests that the TrpRS-mediated high-affinity Trp transport mechanism is mainly Na^+^-independent.

### 3.2. Overexpression of Human IDO1 in HeLa Cells Significantly Stimulates High-Affinity Trp Uptake upon Addition of Purified TrpRS Protein

**Figure 2:**
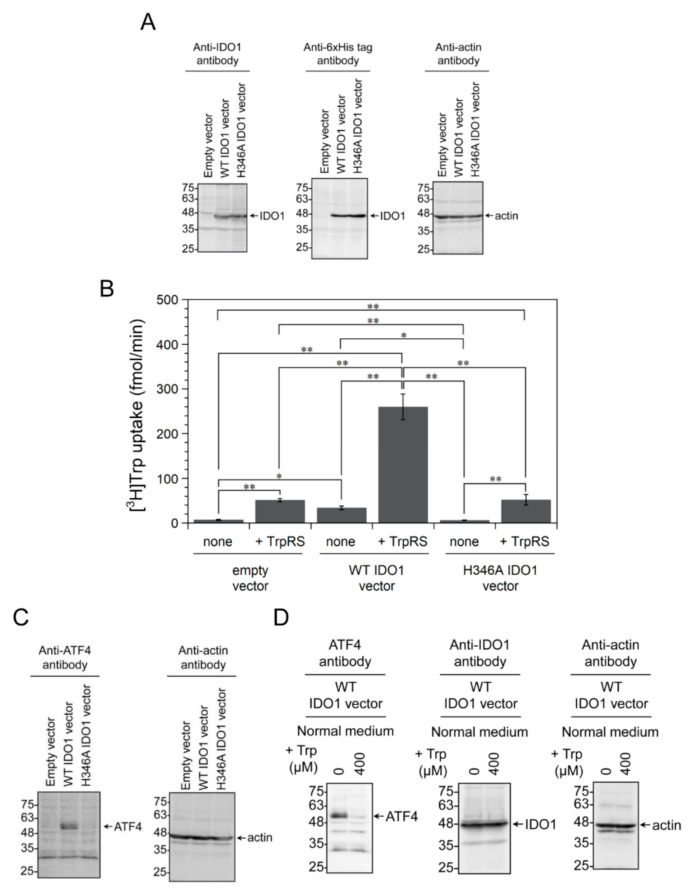
The effect of adding human TrpRS protein to the assay buffer on [^3^H]Trp uptake into HeLa cells overexpressing 6×His-tagged human indoleamine 2,3-dioxygenase 1 (IDO1). Control vector, human wild-type (WT) IDO1 or H346A IDO1 mutant expression vector was transfected into cells. (**A**) Western blot analysis of extracts from 6×His-tagged human IDO1-overexpressing cells using anti-IDO1, anti-6×His tag, and anti-β-actin antibodies. The arrows indicate the expected position of IDO1 and β-actin. Molecular size markers (kDa) are shown on the left. (**B**) [^3^H]Trp uptake into HeLa cells, in which human WT IDO1 or H346A IDO1 mutant was overexpressed, in the absence or presence of human TrpRS protein. All data are expressed as mean ± SD of at least four independent experiments. ** p < 0.01, * p < 0.05. (**C**) Western blot analysis of extracts from 6×His-tagged human IDO1-overexpressing cells using anti-activating transcription factor 4 (ATF4) or anti-β-actin antibodies. The arrows indicate the expected positions of ATF4 and β-actin. Molecular size markers (kDa) are shown on the left. (**D**) Western blot analyses of extracts of 6×His-tagged human WT IDO1-overexpressing HeLa cells, which were incubated in normal medium with additional Trp (0 or 400μM Trp) for 18 h, by using anti-ATF4, anti-IDO1, and anti-β-actin antibodies. Final concentration of Trp in the medium is 80 or 480 μM, because normal medium contains 80 μM Trp.

Our results show that extracellular TrpRS induces high-affinity Trp uptake. However, the relationship between TrpRS and IDO1 in high-affinity Trp uptake has not been clarified. Therefore, in order to verify whether the expression of IDO1 affects high-affinity Trp uptake upon addition of extracellular TrpRS, we tried to measure Trp uptake upon addition of TrpRS protein to the assay buffer of IDO1-overexpressing cells. Initially, we transfected a human IDO1 expression vector into HeLa cells. Overexpression of 6×His-tagged human wild-type (WT) IDO1 (predicted molecular mass: 48 kDa) in HeLa cells was verified by Western blotting using anti-IDO1 and anti-6×His tag antibodies (Figure 2A). β-actin was employed as a loading control (Figure 2A). Overexpression of human WT IDO1 protein in HeLa cells increased Trp uptake rates into the cells compared to cells transfected with empty vector (Figure 2B). IDO1 is a hemoprotein and the proximal heme ligand of human IDO1 is His346 [42]. We prepared the H346A IDO1 mutant, which is unable to bind heme and catabolize Trp to Kyn [43]. Expression of this mutant protein in HeLa cells was confirmed by Western blotting (Figure 2A). Next, we investigated the effect of overexpressing the H346A IDO1 mutant on high-affinity Trp uptake. Our findings showed that the H346A IDO1 mutant did not enhance Trp uptake into HeLa cells (Figure 2B), unlike WT IDO1. These results are in accordance with our previous paper which demonstrated that the expression of human WT IDO1, but not H346A IDO1, induces high-affinity Trp uptake [4].

Next, we tried to investigate the effect of extracellular TrpRS protein on [^3^H]Trp uptake by IDO1-overexpressing cells. As a control, we transfected empty vector into the cells. The addition of TrpRS protein to the assay buffer stimulated Trp uptake (Figure 2B). We subsequently used human WT IDO1-overexpressing cells in the analysis. As shown in Figure 2B, when TrpRS was added to the assay buffer of WT IDO1-overexpressing cells, a marked increase in Trp uptake was observed compared to cells without addition of TrpRS or with empty vector-transfected cells to which TrpRS was added. To verify whether the Trp metabolic activity of IDO1 is important for this increase in Trp uptake, we utilized the H346A IDO1 mutant. As shown in Figure 2B, the expression of H346A IDO1 had no effect on TrpRS-mediated Trp uptake by cells in comparison to HeLa cells expressing WT IDO1 to which TrpRS was added.

It has been reported that the expression of IDO1 in cells causes Trp starvation, leading to the increase of protein levels of activating transcription factor 4 (ATF4) that respond to Trp withdrawal [44,45]. We next investigated the expression levels of ATF4 and β-actin proteins in HeLa cells in which human WT IDO1 was overexpressed, by Western blot analyses using anti-ATF4 or anti-β-actin antibodies. β-actin was employed as a loading control. As shown in Figure 2C, WT IDO1-overexpressing cells upregulated the expression of ATF4, whereas H346A IDO1 mutant-overexpressing cells did not. Moreover, supplementation of Trp to normal culture medium of WT IDO1-overexpressing cells suppressed the induction of ATF4 expression (Figure 2D). These results suggest that stress responses resulting from Trp depletion are induced in IDO1-overexpressing cells.

### 3.3. Trp Depletion, but Not Kyn Production, Enhances Extracellular TrpRS-Mediated High-Affinity Trp Uptake

As shown in Figure 2, overexpression of IDO1 significantly enhanced extracellular TrpRS-mediated high-affinity Trp uptake. Based on these observations, it is possible that either the Trp metabolite generated by IDO1 results in high-affinity Trp uptake, or that the Trp-deficient state of cells caused by Trp consumption by IDO1 induces high-affinity Trp uptake.

**Figure 3:**
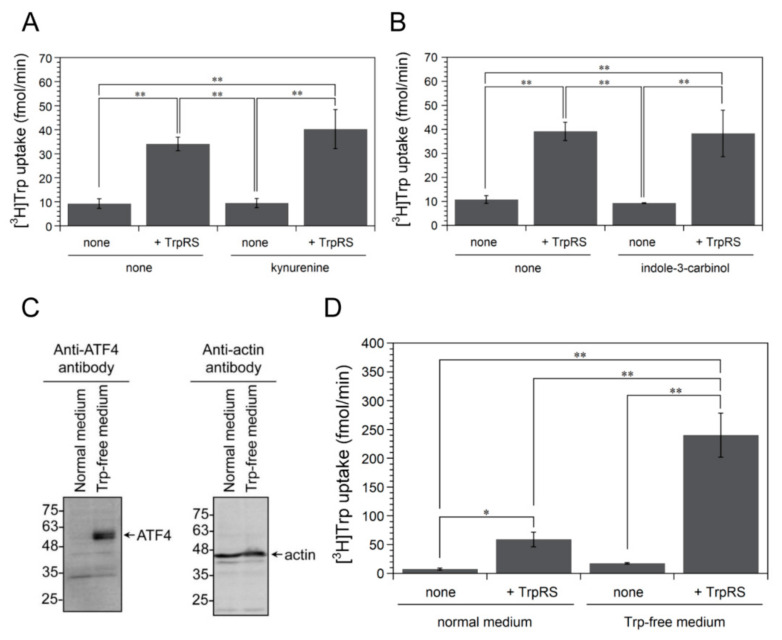
The effect of the aryl hydrocarbon receptor (AhR) agonists or Trp depletion on TrpRS-mediated high-affinity Trp uptake. (**A**) [^3^H]Trp uptake into HeLa cells, which were treated without or with 1 mM kynurenine (Kyn) for 24 h, in the absence or presence of human TrpRS protein. All data are expressed as means ± SD of three independent experiments. ** p < 0.01. (**B**) [^3^H]Trp uptake into HeLa cells, which were treated with 0.05% dimethyl sulfoxide (DMSO) as a control or 50 μM indole-3-cabinol (I3C) dissolved in DMSO (final concentration 0.05%) for 24 h, in the absence or presence of human TrpRS protein. All data are expressed as means ± SD of three independent experiments. ** p < 0.01. (**C**) Western blot analysis of extracts from HeLa cells incubated in normal medium or in Trp-free medium. Western blot analysis of HeLa cell extracts using anti-ATF4 or anti-β-actin antibodies. The arrows indicate the expected positions of ATF4 and β-actin. Molecular size markers (kDa) are shown on the left. (**D**) [^3^H]Trp uptake into HeLa cells, which were incubated in normal medium or in Trp-free medium, in the absence or presence of human TrpRS protein. All data are expressed as mean ± SD of four independent experiments. ** p < 0.01, * p < 0.05.

Previous studies have reported that Kyn generated by IDO1 Trp metabolism activates aryl hydrocarbon receptors (AhR) and increases cellular Trp uptake [3]. Kyn is a physiological AhR agonist, whereas indole-3-carbinol (I3C) acts as a pharmacological AhR agonist [3,46]. Initially, we incubated HeLa cells in the absence or presence of Kyn or I3C for 24 h, and investigated the effects of AhR agonists on high-affinity Trp uptake mediated by extracellular TrpRS. As shown in Figure 3A, B, high-affinity Trp uptake mediated by TrpRS into HeLa cells treated with Kyn or I3C was very similar to the levels in control nontreated cells, suggesting that AhR agonists do not play an important role in high-affinity Trp uptake mediated by extracellular TrpRS.

Next, in order to investigate whether Trp depletion via IDO1 is crucial for Trp uptake, we tried to use HeLa cells, which are incubated in artificial synthetic cell culture medium lacking Trp, as a simplified model of Trp-depleted HeLa cells, in which IDO1 protein was overexpressed. At first, we investigated the expression levels of ATF4 and β-actin proteins in HeLa cells which were incubated in normal or Trp-free cell culture medium, by Western blot analyses using anti-ATF4 or anti-β-actin antibodies. β-actin was employed as a loading control. As shown in Figure 3C, treatment of HeLa cells in Trp-free medium induced an increase in the expression level of human ATF4 protein. Next, we incubated HeLa cells in normal or Trp-deficient cell culture medium for 24 h and examined the effect of adding human TrpRS protein on [^3^H]Trp uptake. As shown in Figure 3D, when TrpRS protein was added to Trp-starved cells, a significant increase in high-affinity Trp uptake was detected, as observed with cells expressing IDO1. Taking these results together, we can conclude that the Trp-deficient state of cells is important for high-affinity Trp uptake into cells.

### 3.4. Overexpression of Human TDO2 in HeLa Cells Enhances Purified TrpRS Protein-Mediated High-Affinity Trp Uptake

Related experiments to those described above were performed with HeLa cells expressing another Trp-metabolizing enzyme, namely TDO2. TDO2 is a heme-binding protein similar to IDO1 that metabolizes Trp to Kyn and is mainly expressed in the liver. Akin to IDO1, it has been suggested that TDO2 metabolism contributes to tumor immune evasion by Trp metabolism [36,37]. His328 of human TDO2 is the heme-binding residue [47] and consequently the H328A TDO2 mutant cannot bind heme and convert Trp to Kyn [48].

**Figure 4:**
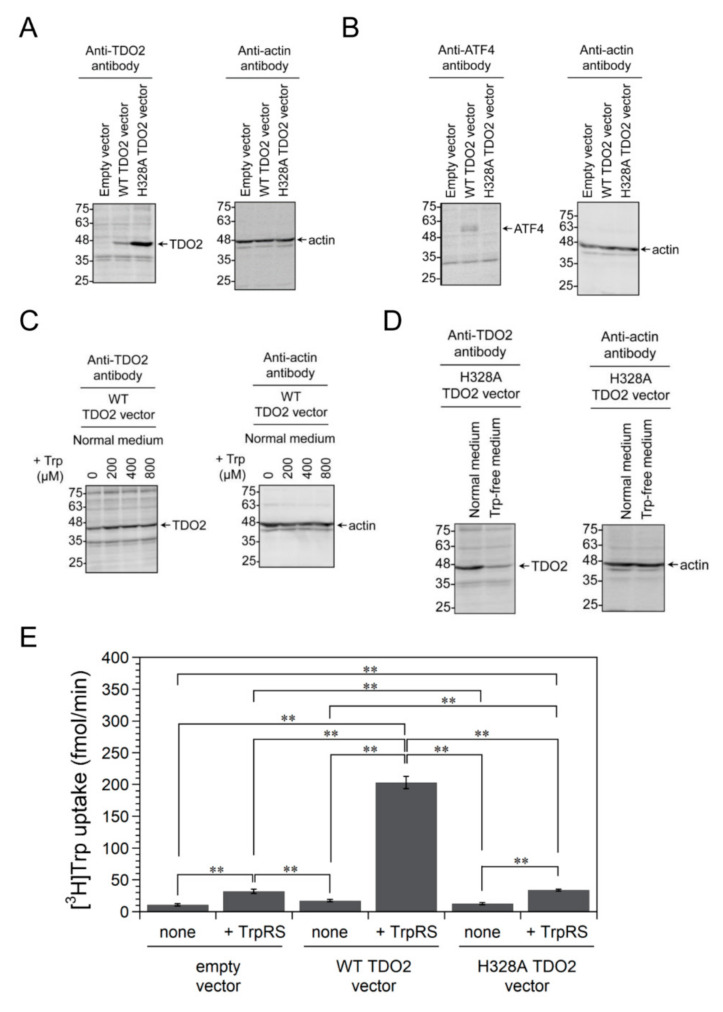
The effect of adding human TrpRS protein to the assay buffer on [^3^H]Trp uptake by HeLa cells overexpressing nontagged human tryptophan 2,3-dioxygenase (TDO2). Control vector, human WT TDO2 or H328A TDO2 mutant expression vector was transfected into HeLa cells. (**A**) Western blot analysis of extracts from nontagged human TDO2-overexpressing cells using anti-TDO2 or anti-β-actin antibodies. The arrows indicate the expected position of TDO2 or β-actin. Molecular size markers (kDa) are shown on the left. (**B**) Western blot analysis of extracts from nontagged human TDO2-overexpressing cells using anti-ATF4 or anti-β-actin antibodies. The arrows indicate the expected positions of ATF4 and β-actin. Molecular size markers (kDa) are shown on the left. (**C**) Western blot analyses of extracts of nontagged human WT TDO2-overexpressing HeLa cells, which were incubated in normal medium with additional Trp (0, 200, 400, or 800 μM Trp) for 18 h, by using anti-TDO2 or anti-β-actin antibodies. Final concentration of Trp in the medium is 80, 280, 480, or 880 μM, because normal medium contains 80 μM Trp. (**D**) Western blot analyses of extracts of nontagged human H328A TDO2 mutant-overexpressing HeLa cells, which were incubated in normal medium or in Trp-free medium for 24 h, by using anti-TDO2 or anti-β-actin antibodies. (**E**) [^3^H]Trp uptake into HeLa cells, in which human WT TDO2 or H328A TDO2 mutant was overexpressed, in the absence or presence of human TrpRS protein. All data are expressed as means ± SD of four independent experiments. ** p < 0.01.

As shown in Figure 4A, overexpression of nontagged human TDO2 (predicted molecular mass: 48 kDa) in HeLa cells was verified by Western blotting using anti-TDO2 antibody. We next investigated the expression levels of ATF4 and β-actin proteins in HeLa cells in which human WT TDO2 was overexpressed, by Western blot analyses using anti-ATF4 or anti-β-actin antibodies. As shown in Figure 4B, WT TDO2-overexpressing cells, but not H328A TDO2 mutant-overexpressing cells, upregulated the expression of ATF4 (Figure 4B), suggesting that stress responses resulting from Trp depletion are induced in WT TDO2-overexpressing cells.

Western blot analysis showed that the amount of WT TDO2 protein is significantly lower than that of H328A TDO2 (Figure 4A). Human TDO2 protein has been reported to be rapidly degraded via ubiquitin-dependent proteasomal degradation (UPD) [47]. Intriguingly, binding of Trp to the exo site of human TDO2 stabilizes human TDO2 against UPD, thereby regulating the half-life of the enzyme [47]. Therefore, the amount of WT TDO2 protein may be decreased relative to H328A TDO2 mutant because of the low concentration of Trp resulting from its catalytic activity. In fact, as shown in Figure 4C, the supplementation of Trp to normal culture medium of WT TDO2-overexpressing cells increased the amount of WT TDO2 protein. On the other hand, incubation of human H328A TDO2 mutant-overexpressing cells in Trp-free medium decreased the amount of H328A TDO2 protein compared to that in normal medium (Figure 4D). These results suggest that TDO2 protein is degraded at low concentrations of Trp.

When TrpRS protein was added to uptake assay buffer of human WT TDO2-overexpressing cells, a marked increase in Trp uptake was observed, as was the case for WT IDO1-expressing cells (Figure 4E). We next investigated the effect of overexpressing H328A TDO2 mutant on Trp uptake. Addition of TrpRS protein to uptake assay buffer of cells overexpressing the H328A TDO2 mutant had no significant effect on high-affinity Trp uptake (Figure 4E).

## 4. Discussion

**Figure 5:**
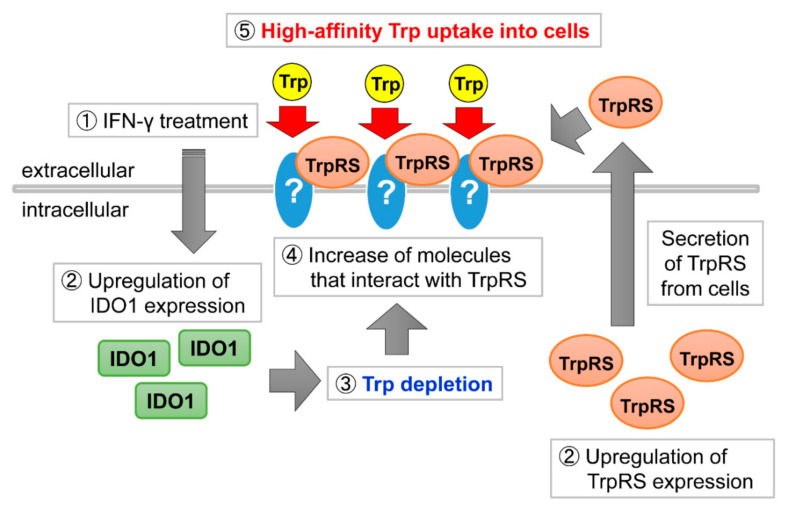
A schematic model of the regulatory mechanism of TrpRS-mediated Trp uptake upon interferon (IFN)-γ treatment of human cells.

Here, we demonstrated that in cells overexpressing the Trp catabolizing protein IDO1 or TDO2 or in Trp-starved cells there is a significant increase in high-affinity Trp uptake upon the addition of purified TrpRS protein to the assay buffer. Based on our experimental results, we propose a model for the mechanism of high-affinity Trp uptake induced by IFN-γ treatment (see Figure 5). In this model, IFN-γ initially stimulates upregulation of IDO1 and TrpRS expression in human cells [4,17,18,19,20,21,22,23,24,25]. Increased expression of IDO1 promotes Trp metabolism, leading to Trp deficiency. Intracellular Trp depletion may increase the expression level of cell-surface molecules that interact with extracellular TrpRS. Earlier studies have shown that TrpRS is secreted and that extracellular TrpRS can be detected in the cell culture medium [32,34,35]. Furthermore, a recent report showed that TrpRS is rapidly secreted from cells following infection with a pathogen [32]. This mechanism appears to constitute a primary defense to protect cells against infection [32]. Extracellularly secreted TrpRS then binds to extracellular Trp and may induce high-affinity Trp uptake that selectively transports Trp into cells via the TrpRS-interacting protein whose expression level is increased.

In the present study, we showed that overexpression of TDO2 in cells has the same effect as IDO1 on TrpRS-mediated high-affinity Trp uptake. Although IDO1 and TDO2 catalyze the same reaction, they have distinct enzymatic characteristics [18,49,50,51] and their amino acid sequence similarity is very low. IDO1 is also a monomeric enzyme, whereas TDO2 forms a homotetramer. Furthermore, protein crystallography studies have shown that the overall structure and spatial orientation of the small domains of the proteins differ in human IDO1 and TDO2, although the large domain of IDO1 exhibits high structural similarity to that of TDO2 [42,47,51]. Given the low level of similarity between IDO1 and TDO2, the present results suggest that protein–protein interactions of IDO1 or TDO2 are not important for high-affinity Trp uptake.

AhR activation mediated by the IDO1/TDO2 product, Kyn, results in the generation of immune-tolerant dendritic cells and regulatory T cells [46,52,53]. Binding of Kyn to AhR on effector T cells suppresses the activity of the effector T cells, helping tumors evade the immune response [46,52]. Bhutia et al. [3] showed that the activation of AhR by AhR agonists, including Kyn, enhanced Trp uptake. These authors proposed that IDO1 may induce expression of Trp transport system via activation of AhR by Kyn [3]. However, in the present study, we demonstrated that the AhR agonists, Kyn and I3C, have no effect on high-affinity Trp uptake. Based on these data, we speculate that the activation of AhR by Kyn may be related to other process of high-affinity Trp uptake, such as secretion of TrpRS from cells. Moreover, we showed that the addition of TrpRS to the assay buffer of Trp-starved cells leads to a significant increase in high-affinity Trp uptake. Taken together, we can conclude that Trp depletion, but not Kyn-AhR signaling, is crucial for high-affinity Trp uptake mediated by extracellular TrpRS protein.

ATF4 is a transcription factor whose expression is increased by amino acid starvation [54,55,56]. It has been reported that Trp depletion activates the general control non-derepressible 2 (GCN2)-ATF4 signal pathway that respond to amino acid withdrawal [44,45,55,57,58]. GCN2 is a kinase that senses amino acid deficiency through binding to uncharged tRNA. Trp depletion activates the stress kinase GCN2, that phosphorylates and downregulates the α-subunit of eukaryotic translation initiation factor 2 (eIF2α), which is a crucial translation initiation factor. Although phosphorylated eIF2α prevents the ribosomal translation of the majority of mRNA species, it upregulates the translation of a select group of transcripts. An example of GCN2-inducible translation is ATF4. The mRNA of ATF4 comprises two upstream open reading frames (uORFs). The second uORF competes with the ATF4 coding sequence for translation initiation [54,55,56]. Phosphorylation of eIF2α mediated by GCN2 results in the second uORF being bypassed [54,55,56]. As a consequence, ATF4 translation is markedly increased [54,55,56]. In the present study, we demonstrated that the expression level of ATF4 is enhanced in cells incubated in Trp-free medium and in IDO1- or TDO2-overexpressing cells, confirming that these latter cells are starved of Trp. These findings suggest that Trp starvation stress triggers a significant increase of TrpRS-mediated high-sensitivity Trp uptake.

Recently, it has been reported that human TrpRS promotes enterovirus (EV)-A71 intracellular entry [59,60]. EV-A71 is a virus that causes hand-foot-and-mouth disease and can cause serious neuropathy. The authors report that upon IFN-γ stimulation TrpRS translocates extracellularly or to the cell membrane, and that extracellular TrpRS binds to EV-A71 and promotes its entry into cells [59,60]. Given that these results are similar to those presented in this study insofar as extracellular TrpRS is involved in the uptake of an extracellular molecule, TrpRS-mediated intracellular entry of EV-A71 may be correlated with the TrpRS-mediated pathway for high-affinity Trp uptake. Therefore, further study of TrpRS-mediated high-affinity Trp uptake may contribute to the identification of new therapeutic targets for hand-foot-and-mouth disease.

## 5. Conclusions

In conclusion, we demonstrated that expression of IDO1 or TDO2 in cells enhances TrpRS-mediated high-affinity Trp uptake into cells. Moreover, we showed that Trp depletion stimulates TrpRS-mediated high-affinity Trp uptake. Further studies are required to identify which interacting molecules are induced by Trp deficiency caused by the Trp-metabolizing enzymes IDO1 and TDO2. In addition, it is also important to clarify the mechanism of extracellular secretion of TrpRS and to elucidate the molecular details of high-affinity Trp uptake via TrpRS.
